# Depressive symptoms during the perinatal period by disability status: Findings from the United States Pregnancy Risk Assessment Monitoring System

**DOI:** 10.1111/jan.15482

**Published:** 2022-11-01

**Authors:** Jeanne L. Alhusen, Rosemary B. Hughes, Genevieve Lyons, Kathryn Laughon

**Affiliations:** 1 University of Virginia School of Nursing, Charlottesville, Virginia, USA; 2 University of Montana Rural Institute for Inclusive Communities, Missoula, Montana, USA; 3 Department of Public Health Sciences, University of Virginia, Charlottesville, Virginia, USA

**Keywords:** antepartum depression, disability, nurses, postpartum depression, pregnancy

## Abstract

**Aims:**

The aim of the current study was to compare the prevalence of depressive symptoms during the perinatal period among respondents with a disability as compared to those without a disability.

**Design:**

We conducted a secondary analysis of nationally representative data from the Pregnancy Risk Assessment Monitoring System data from 24 participating United States between 2018 and 2020.

**Methods:**

A cross-sectional sample of 37,989 respondents provided data on disability, including difficulty in vision, hearing, ambulation, cognition, communication and self-care. The outcome of interest was perinatal depressive symptoms, defined as experiencing depressive symptoms during the antenatal period or postpartum period. Regression models were used to calculate odds of depressive symptoms during these two time periods by disability status while controlling for relevant sociodemographic characteristics and depressive symptoms prior to pregnancy.

**Results:**

Respondents with disabilities experienced a higher prevalence of depressive symptoms in both the antenatal period and postpartum period as compared to those without disabilities. In fully adjusted models, respondents with disabilities had 2.4 times the odds of experiencing depressive symptoms during pregnancy and 2.1 times the odds of experiencing postpartum depressive symptoms as compared to respondents without disabilities.

**Conclusion:**

Respondents with disabilities experience a higher prevalence of depressive symptoms throughout the perinatal period thereby increasing the risk for adverse maternal, neonatal and infant health outcomes.

**Impact:**

Perinatal depression is a significant public health issue globally, and our findings suggest that persons with disability are at an increased risk for depressive symptoms both during pregnancy and in the postpartum period. Our findings represent a call to action to improve clinical and supportive services for women with disabilities during the perinatal period to improve their mental health and the consequent health of their offspring.

## 1 | INTRODUCTION

Perinatal depression, a highly prevalent and complex complication of pregnancy and childbirth, is associated with multiple adverse maternal and child outcomes (ACOG Committee Opinion No. 757, 2018). Encompassing antenatal depression that occurs during pregnancy and postpartum depression occurring in the first 12 months following birth, perinatal depression is experienced by one in seven women (ACOG Committee Opinion No. 757, 2018). Although 11.5% has long been cited as the mean prevalence of perinatal depression (Gavin et al., 2005), rates vary depending on the measurement method and the period on which prevalence is measured. For example, a 2005 systematic review (Gaynes et al., 2005) revealed estimates for antenatal depression ranging from 8.5% to 11.0% and 6.5% to 12.9% for postpartum depression. However, a 2016 literature review yielded an average prevalence of 17% for antenatal depression and 13% for postpartum depression (Underwood et al., 2016). According to an analysis of 2018 data from the Pregnancy Risk Assessment Monitoring System (PRAMS) administered in 31 states, the average prevalence of postpartum depression was 13.2%, ranging from 9.7% to 23.5%, depending on the state reporting (Bauman et al., 2020). That study also reported on the prevalence of women having been asked about depression by a provider during prenatal care (79.1%) and postpartum visits (87.4%).

## 2 | BACKGROUND

The aetiology of perinatal depression is complex involving sociodemographic, psychosocial and biological risk factors (Dagher et al., 2021). Prominent documented risks for both antenatal and postpartum depression include low socioeconomic status, single marital status, prior or current intimate partner violence, history of depression and/or anxiety, adverse life events, history of adverse childhood experiences, poor social support, unintended pregnancy, gestational diabetes and racial/ethnic minority status (Dagher et al., 2021; Liu et al., 2013; Mukherjee et al., 2016; Wilson et al., 2020). Risks identified for depression during pregnancy include pregnancy complications, poor relationship quality and receipt of public health insurance (Dagher et al., 2021). Risk factors specific for postpartum depression include prenatal and antenatal depression and anxiety, low self-esteem, childcare stress, obstetric complications, postpartum blues, difficult infant temperament, sudden change in reproductive hormones following birth and genetic variations (Dagher et al., 2021; Guintivano et al., 2018). The literature is mixed regarding maternal age, with some research suggesting the risk for postpartum depression is greater under age 24, decreases between ages 24 and 35, but increases with advanced maternal age (Guintivano et al., 2018).

When untreated, perinatal depression can contribute to multiple negative maternal and child consequences. Depression during pregnancy and postpartum is associated with maternal suicidal ideation, breastfeeding difficulties, paternal depression and child behavioural and mental health problems (Alhusen et al., 2013; Dagher et al., 2021; Field, 2017; Letourneau et al., 2012; Slomian et al., 2019). Documented consequences of antenatal depression include maternal sleep problems, depressive symptoms progressing to postpartum depression, obesity, gestational diabetes, preterm delivery, preeclampsia, low birth weight, lower maternal sensitivity and negative perceptions of infant temperament (Alhusen et al., 2016; Cattane et al., 2021; Dagher et al., 2021; Field, 2017; Mukherjee et al., 2016). Postpartum depression is associated with maternal and infant health problems; relationship, family functioning and parenting difficulties; maternal substance misuse; poor maternal attachment and developmental problems in the child (Alhusen et al., 2021; Dagher et al., 2021; Goodman, 2019; Slomian et al., 2019). When severe, postpartum depression can be associated with suicidal ideation, thoughts of infant harm and in rare cases, infanticide (Van Niel & Payne, 2020).

Perinatal depression may be particularly salient for the approximate 12% of U.S. women of childbearing age who have a disability (Courtney-Long et al., 2015). Women with disabilities are increasingly choosing to become pregnant and give birth, with pregnancy occurrence similar to women without disabilities (10.8% vs. 12.3%) (Horner-Johnson et al., 2016). Although many women with disabilities experience successful pregnancies, growing evidence links maternal disability with a risk for adverse maternal and neonatal outcomes such as a greater risk of pregnancy complications and perinatal depression (Gleason et al., 2021; Mitra, Parish, et al., 2015; Signore et al., 2011).

Depression and other psychological distress are disproportionally prevalent in people with disabilities (Cree et al., 2020). Women with disabilities are at a greater risk for depression than women without disabilities, men with disabilities, and the general population (Chevarley et al., 2006). Multiple studies based largely on convenience samples demonstrate that women with disabilities are at an elevated risk for perinatal depression (Brown et al., 2016, 2017; Krysko et al., 2022; Pohl et al., 2020), a risk confirmed by population-based research in Norway, Canada and the United States (Brown et al., 2019; Eid et al., 2021; Mitra, Parish, et al., 2015).

Analysing data from the Rhode Island PRAMS, Mitra and colleagues reported higher rates of postpartum depression in mothers with disabilities (28.9%) than those without disabilities (10%) (Mitra, Parish, et al., 2015). This disparity was observed after accounting for sociodemographic variables, other maternal characteristics linked with postpartum depression, prenatal and antenatal depression and seeing a provider for mental health issues. Although greater antenatal depression was observed in women with versus without disabilities (25.2% and 7.6% respectively), it was only marginally associated with postpartum depression in women with disabilities. Women with disabilities’ risk factors for postpartum depression included medical problems or intimate partner violence during pregnancy. When compared to other mothers, those with disabilities were more likely to disclose perinatal depression symptoms to their healthcare provider. Although representing the first population-based investigation of postpartum depression in women with disabilities, it was limited to data collected in only one state. No known research has used representative data from multiple states to estimate the prevalence of perinatal depression in women with disabilities.

## 3 | THE STUDY

### 3.1 | Aims

The purpose of the current study was to examine the prevalence of antenatal and postpartum depression among women with and without disabilities who have given birth through the analysis of a PRAMS dataset which recently incorporated a disability questionnaire supplement (Svestkova, O., 2008).

### 3.2 | Design and setting

For the current analyses, we analysed data from CDC’s PRAMS, a surveillance system that collects data from the time before, during and shortly after pregnancy among persons who have recently given birth. The goal of the PRAMS project is to improve the health of mothers and infants by reducing adverse outcomes. As such, PRAMS collects information on maternal attitudes and experiences throughout the perinatal period including attitudes and feelings about the most recent pregnancy, preconception care, breastfeeding intention and duration, cigarette smoking and alcohol use, abuse during the perinatal period, mental health, infant health care and contraceptive use. Potential respondents are contacted between 2 and 6 months postpartum by mail through a pre-letter that introduces PRAMS to the respondent and informs her that a questionnaire will soon arrive. This packet is sent to all sampled respondents 3–7 days after the pre-letter. Additionally, a tickler note is sent with up to two additional questionnaire packets sent for respondents who do not respond to initial mailings. If no response by mail, they are contacted by telephone. Per CDC guidelines, data are available for states meeting a minimum response rate of greater than or equal to 55%. On average, 75% of states have met or exceeded the threshold since 2007. Survey responses are linked to birth certificate data for analyses. We used data from 2018 to 2020, the most recent data available at the time of data analysis.

### 3.3 | Respondents

The population of interest for each PRAMS state is resident women who recently gave birth within their state to a live-born infant during the surveillance year. Annual sample sizes per state range from 1000 to 3000 respondents. Sample sizes are determined according to stratification plan, number of births and available budgets. A state’s birth certificate file serves as the sampling frame for identifying new mothers. To ensure that women with multiple births are sampled at the same rate as those with singleton births, only one infant from a multiple gestation is randomly selected to be included in the sampling frame. The PRAMS sample is stratified so that subpopulations of particular public health importance can be oversampled, including mothers of low birthweight infants, those living in high-risk geographic locations, and racial and ethnic minority groups. Individual states and territories choose a stratification plan based on their public health priorities.

The sample for this analysis included respondents who had a live birth from 2018 to 2020 and were asked the Washington Group Short Set of Questions on Disability (WG-SS) disability questions (unweighted *n* = 45,561). The WG-SS questions were asked in 24 states, with some states discontinuing (Maine, New York, Rhode Island, West Virginia) and other states (Tennessee, New Hampshire) incorporating WG-SS disability questions during the study period. We carefully analysed the WG-SS responses by state, by year and by ‘batch’ for completion; our sample included only responses from batches in which the WG-SS questions were asked. Participants who were not asked the WG-SS questions were excluded from our sample, and participants who were not asked about depressive symptoms were excluded from our sample.

**FIGURE 1:**
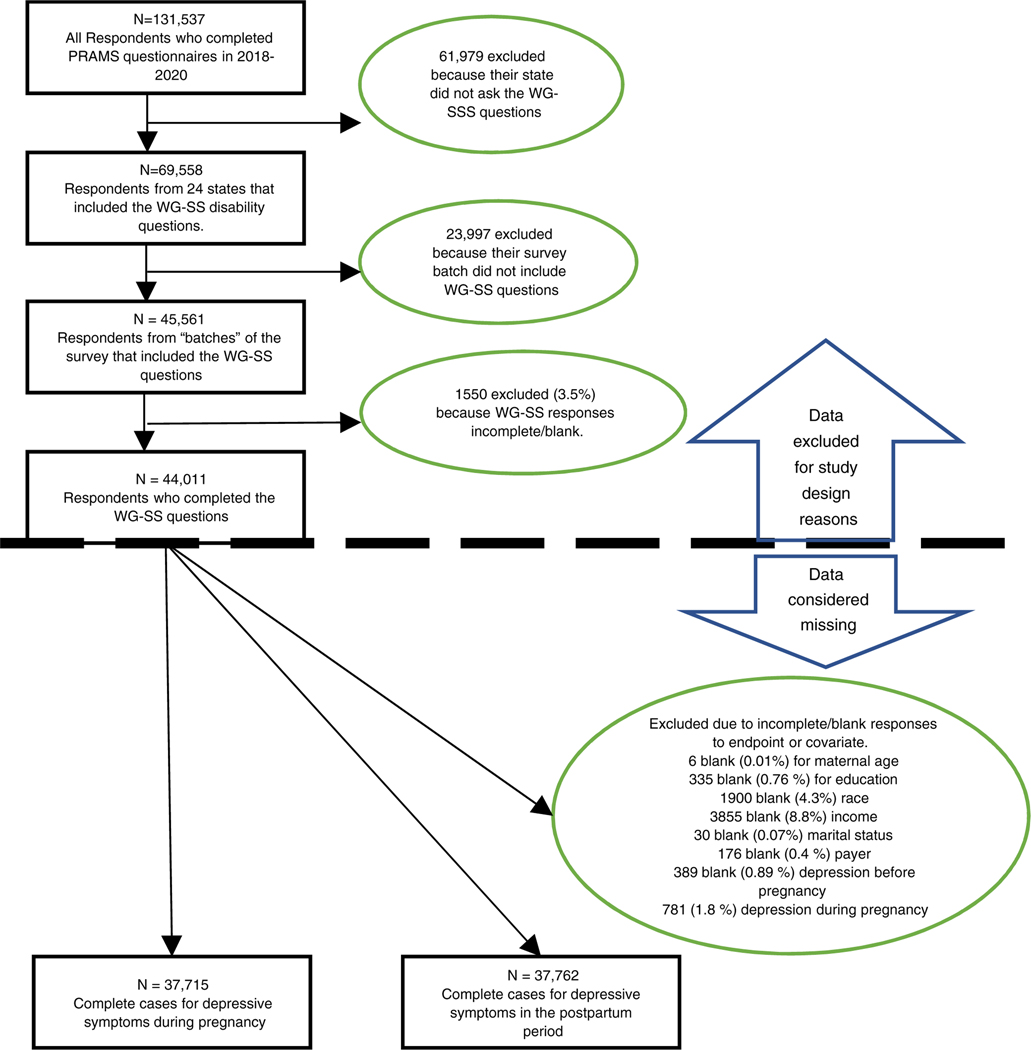
Participant inclusion flow chart

Overall, 24 states were included in our sample. Regarding missing data, 781 participants (1.7%) had missing responses for depressive symptoms during pregnancy, and 989 participants (2.2%) had missing responses for depressive symptoms during the postpartum period. Other covariates were between 0% and 8.5% missing (see Figure 1), thus we performed complete case analysis. This resulted in 37,715 complete cases for depressive symptoms during pregnancy and 37,762 complete cases for depressive symptoms in the postpartum period.

### 3.4 | Data collection

In 2018, a series of questions related to disability were added as an optional questionnaire for participating states and territories. The disability questionnaire supplement consists of the WG-SS that has been used within other federal and global surveys. These questions are based on the World Health Organization’s International Classification of Functioning, Disability and Health and provide standardized language as well as a framework for operationalizing disability (Svestkova, O., 2008). Respondents are asked five disability questions including if they have difficulty seeing, even when wearing glasses or contact lenses; difficulty hearing, even if using a hearing aid(s); difficulty walking or climbing steps; difficulty remembering or concentrating; difficulty with self-care, such as washing or dressing and difficulty communicating, understanding or being understood in their usual language. Response options include no difficulty, some difficulty, a lot of difficulty and cannot do this at all. Aligned with recommendations of subject matter experts at the National Institutes of Health and CDC, and in consultation with the CDC PRAMS teams, we coded a response of ‘no difficulty’ or ‘some difficulty’ as ‘no disability’ while responses of ‘a lot of difficulty’ or ‘I cannot do this at all’ were coded as ‘yes disability’. Respondents who answer ‘no’ to some disability questions and leave one or more of the other disability questions blank are considered missing data. The disability questions were first asked in 2018 in 22 states, but not in all ‘batches’ or months of PRAMS administration.

### 3.5 | Ethical considerations

Prior approval for this study was obtained from the institutional review board of the study team’s institution. As analyses included publicly available, de-identified surveillance data, this study was considered exempt. Permission was also received from the PRAMS working group.

### 3.6 | Data analysis

The outcomes of interest included depression during pregnancy and in the postpartum period. Depression during pregnancy was assessed using the question ‘During your most recent pregnancy, did you have any of the following conditions? …Depression’ (yes or no). Respondents self-report ‘yes’ or ‘no’ and this is not corroborated with a medical diagnosis. For the postpartum period, respondents were asked ‘Since your new baby was born, how often have you felt down, depressed or hopeless?’ and ‘Since your new baby was born, how often have you had little interest or pleasure in doing things you usually enjoyed?’ Response choices included always, often, sometimes, rarely and never. These two questions were validated for the screening of general depression and were utilized by CDC as a surveillance tool for self-reported postpartum depression on PRAMS. Respondents who responded ‘always’ or ‘often’ to either question were classified as experiencing postpartum depression.

Covariates of interest were selected as potential confounders of the association of disability and depression based on the literature (Cree et al., 2020; Nosek et al., 2008). Covariates obtained from the birth certificate included maternal age, race, ethnicity, educational attainment, marital status and insurance status. Depression prior to pregnancy is also an important covariate. This covariate was ascertained from a question which asks respondents ‘During the 3 months before you got pregnant with your new baby, did you have any of the following conditions? … Depression (yes or no)’.

Depressive symptoms prior to and during pregnancy are highly correlated, thus could not both be included in the postpartum depression model. As such, a composite variable was defined for history of depression. Symptoms were considered present if a respondent had depressive symptoms either before or during pregnancy, and absent if a respondent reported no depressive symptoms in either period. This variable was included in the model for postpartum depression.

All analyses were conducted using the complex survey features of SAS v. 9.4 to account for the sampling process, design and adjusting for nonresponse, and the potential for clustering around particular healthcare facilities, counties or time of year and provide results that are representative of the total population of mothers who gave birth to a live infant in the states/territories and time periods under study. Specifically, SAS PROC SURVEYFREQ was used for estimation of prevalence and confidence intervals, the Rao–Scott chi-square test was used to test for significant differences in prevalence of depressive symptoms by group and PROC SURVEYLOGISTIC was used to run logistic regression models and estimate odds ratios for depression during pregnancy and in the postpartum period with adjustment for covariates. Separate models were estimated for depression during pregnancy and depression during the postpartum period.

### 3.7 | Validity, reliability and rigour

The PRAMS is one of the largest state-based surveillance systems that include women with live births, including their experiences over the perinatal period. Data obtained from PRAMS are linked to birth certificate information. Because PRAMS data are self-reported, the reliability and validity with other population-based data collection systems, such as the birth certificate, have been confirmed in multiple studies (Ahluwalia et al., 2013; Gayle et al., 1988; Hosler et al., 2010). PRAMS incorporates a number of quality control measures. Data entry verification is required for a minimum of 10% of mail surveys received and many states perform 100% verification. Supervisors are required to monitor 10% of all telephone interviews to assure proper survey administration and recording of responses. Nonresponse rates are low (1% to 2% for most questions) with the exception of response rates to the question on household income (averages 6% nonresponse rate). No imputation procedures are used for item nonresponse.

## 4 | RESULTS

The sample included *n* = 35,404 respondents (unweighted) who did not report a disability and *n* = 2585 respondents who reported at least one disability; full characteristics are displayed in Table 1. The groups differed significantly with respect to age, education, race, income, relationship status and insurance provider. Respondents with disabilities were more likely to report obtaining a high school education or less and were significantly more likely to be non-white. The majority (51%) of respondents with a disability reported a household income below the federal poverty level (FPL) as compared to 27% of respondents who did not report a disability. Related, among respondents who reported no disability, 51% had a household income that exceeded 200% of FPL. About 64% of respondents who reported no disability were married as compared to 43% of respondents with at least one disability. Regarding insurance, 55% of respondents with no disability had private insurance while 32% of respondents with at least one disability reported private insurance.

In regard to depressive symptoms, about 14.4% (95% CI 13.8–15.0) of respondents with no disability reported depressive symptoms in the 3 months before pregnancy. In contrast, 43% (95% CI 40.0–46.7) of respondents with at least one disability reported depressive symptoms during the same timeframe. The prevalence of depressive symptoms in the antenatal and postpartum periods is shown in Table 2. During pregnancy, respondents with no reported disability had an estimated prevalence of depressive symptoms of 13.5% (95% CI 12.9–14.1). Respondents with at least one disability reported an estimated prevalence of 43.1% (95% CI 39.8–46.4). For depressive symptoms in the postpartum period, the rates were slightly lower. Among respondents with no disability, approximately 12.1% (95% CI 11.5–12.6) reported depressive symptoms, compared to 33.6% (95% CI 30.4–36.6) among respondents with at least one disability. Statistical comparisons between these groups differ significantly with *p* < .001.

Separate multivariable models were estimated predicting depressive symptoms during pregnancy and in the postpartum period. The during-pregnancy model adjusted for age, education, race, income, relationship status, insurance and depressive symptoms in the 3 months prior to pregnancy. The postpartum model adjusted for age, education, race, income, relationship status, insurance and the composite variable history of depression (defined as having depressive symptoms before or during pregnancy).

The adjusted odds ratio (aOR) for depressive symptoms during pregnancy among respondents with at least one disability was 2.43 (95% CI 1.97–3.0) indicating these respondents had more than double the odds of depressive symptoms compared to those without a disability (Table 3). Other covariates associated with depressive symptoms were Hispanic race (associated with reduced odds of depressive symptoms), income below 200% FPL, relationship status and depressive symptoms in the 3 months prior to pregnancy. Consistent with other studies, depression prior to pregnancy is the most significant risk factor for depressive symptoms during pregnancy (aOR 33.57; 95% CI 29.63–38.03) (Field, 2017; Lancaster et al., 2010).

Regarding depressive symptoms in the postpartum period, respondents with at least one disability had an aOR of 2.14 (95% CI 1.80, 2.54), indicating they had more than twice the odds of postpartum depressive symptoms compared to those reporting no disabilities (Table 4). Other covariates associated with depressive symptoms in the postpartum period were maternal age, education, race, income, having Medicaid insurance and having a history of depression.

## 5 | DISCUSSION

The results of this U.S. population-based analysis reveal that women with disabilities were over twice as likely to experience depressive symptoms during pregnancy and in the postpartum period as compared to those without a disability. These significant differences were noted after controlling for relevant sociodemographic characteristics as well as depression prior to pregnancy. The prevalence of postpartum depressive symptoms in women with disabilities (33.6%) was lower than noted in a recent analysis of Massachusetts PRAMS data (2012–2017) in which 37.4% of women with disabilities reported postpartum depressive symptoms. Yet, the prevalence of postpartum depressive symptoms in women without disabilities was higher in our sample (12.1%) as compared to that noted in Massachusetts PRAMS (8.8%) (Booth et al., 2021). Across both time points, our prevalence was higher than findings of an analysis of Rhode Island PRAMS data (2009–2011) in which approximately 30% of women with disabilities versus 10% of women without disabilities reported depressive symptoms in the postpartum period (Mitra, Iezzoni, et al., 2015). Yet, in a recent analysis of women with multiple sclerosis receiving care at an academic health centre, the prevalence of postpartum depressive symptoms was significantly lower (13%) (Krysko et al., 2022). Authors posited that their results underestimated the true prevalence of postpartum depressive symptoms due to a lack of standardized screening in place. A notable strength of the current study is the standardized measure of depressive symptoms during pregnancy and in the postpartum period across all participating states.

Perinatal depression is a complex phenomenon in the context of disability. First, depressive symptoms also represent common disability-related symptoms (e.g. fatigue or memory problems), which can result in overestimating depression or be mistaken for psychological distress and result in underdiagnosing depression in persons with disabilities. Second, women with disabilities experience a high prevalence of risk factors associated with perinatal depression including a history of depression (Robinson-Whelen et al., 2013), interpersonal violence (Alhusen et al., 2019, 2022; Hughes et al., 2011), unintended pregnancy (Alhusen et al., 2019; Horner-Johnson et al., 2020; Mosher et al., 2018) and socioeconomic disadvantage (Krahn et al., 2015). Additionally, compared to other pregnant women, those with disabilities are at greater risk for severe pregnancy- and birth-related complications (Gleason et al., 2021), which potentially increase their risk for experiencing symptoms of depression around the time of pregnancy. Women with disabilities lack information about reproductive health germane to their disability (Hughes et al., 2022). They also express unique concerns about pregnancy and motherhood which may increase their vulnerability to perinatal depression. For example, women with disabilities may have well-grounded apprehension that caesarean section decisions will be made based on the presence of disability and in the absence of medical necessity (Gleason et al., 2021). They may also be discouraged from becoming pregnant due to health concerns, and experience stigmatizing societal reactions about their pregnancy (Alhusen et al., 2020; Iezzoni et al., 2015).

Women with disabilities also face multiple unremitting barriers to receiving equitable care including physical or health system barriers such as inaccessible medical facilities and equipment, lack of interpreter services and other communication barriers, financial limitations and widespread discriminatory attitudes and biases of providers who question their ability for pregnancy, childbirth and parenting (Saeed et al., 2022; Tarasoff, 2017). Moreover, clinical tools may not be accessible to them. For example, the U.S. Preventive Services Task Force, the American College of Obstetricians and Gynaecologists and multiple other professional associations recommend depression screening as an evidence-based standard of perinatal care for all women (ACOG Committee Opinion No. 757, 2018). Although screening is a critical first step to guiding treatment for women with perinatal depression, validated screening tools (e.g. Edinburgh Postnatal Depression Scale, Patient Health Questionnaire) may not be accessible to persons with disability due to disparities in English literacy, health literacy or cognitive limitations. These important barriers are not routinely assessed in population-based datasets such as PRAMS.

Perinatal depression confers significant risk to mothers, infants and families. Our results have important implications for the development and tailoring of support for women with disabilities, including addressing mental health risks before pregnancy and providing comprehensive care across the perinatal period. Preconception care for women with disabilities should be responsive to the myriad ways disability shapes healthcare access and receipt of care as well as how disability interacts with other important social determinants of health including poverty and education. Increased clinical and research attention to practices, policies and interventions for improving the health and healthcare access of child-bearing age women with disabilities will help reduce the disproportionately high rates of perinatal depression in this large, underserved and marginalized population of women.

### 5.1 | Limitations

Our findings are subject to several limitations. First, depression during pregnancy and in the postpartum period are self-reported and might not represent a clinical diagnosis of depression. The PRAMS postpartum depression two-item screener is based on the Patient Health Questionnaire-2 (Levis et al., 2020). These questions with similar categorization have a sensitivity of 58% and specificity of 85%, compared with clinical assessments of major depressive episodes (O’Hara et al., 2012). Thus, our results might underestimate the true prevalence of postpartum depression. This appears likely given that in our sample we observed a substantial difference in rates of depressive symptoms before/during pregnancy compared with in the postpartum period. Additionally, self-reported data may be subject to social desirability, recall and other biases. Second, PRAMS has limited data on mental health treatment, including pharmacotherapy use which is an important consideration given noted disparities in mental health access to treatment for persons with disabilities (Roll et al., 2013). Finally, our analysis was limited to respondents who gave birth in the United States. Perinatal depression is a global public health issue (Gavin et al., 2005), and these relationships should be examined in all contexts with the goal of improving mental health and pregnancy outcomes of child-bearing age women with disabilities.

## 6 | CONCLUSION

Our study suggests that women with disabilities are at an increased risk of experiencing depressive symptoms during the perinatal period compared to women without disabilities. Our findings represent a call to action to improve clinical and supportive services for women with disabilities during the perinatal period and to conduct research designed to explore factors contributing to perinatal depression disparities in women with disabilities. Providing focused support around the time of pregnancy and increasing disability-sensitive training for clinicians will have the potential to enhance the perinatal health and health care of women with disabilities.

## Figures and Tables

**TABLE 1:** Respondent characteristics

	No disability reported (unweighted *n* = 35,404, weighted *n* = 1,616,417)		At least one disability (unweighted *n* = 2585, weighted *n* = 109,546)	
Characteristic	Column %	95% CI	Column %	95% CI
Age^a^				
<20years	3.25	(2.92, 3.57)	5.22	(3.74, 6.70)
20–24years	17.22	(16.54, 17.91)	27.48	(24.43, 30.53)
25–34years	60.47	(59.61, 61.33)	53.18	(49.84, 56.52)
35 +years	19.06	(18.39, 19.73)	14.12	(11.88, 16.36)
Education Level^a^				
<High School	9.04	(8.51, 9.58)	15.63	(13.01, 18.25)
High School	24.01	(23.23, 24.79)	36.35	(33.04, 39.66)
Some college	26.03	(25.28, 26.78)	31.89	(28.97, 34.82)
Bachelors+	40.92	(40.09, 41.75)	16.12	(13.71, 18.54)
Race^b^				
Asian non-HS	4.21	(3.88, 4.53)	2.99	(1.97, 4.01)
White non-HS	60.44	(59.63, 61.24)	57.08	(53.78, 60.39)
Black non-HS	17.92	(17.28, 18.56)	21.08	(18.39, 23.78)
Other/Mixed	3.55	(3.26, 3.84)	4.26	(3.17, 5.35)
Hispanic	13.88	(13.28, 14.49)	14.58	(12.06, 17.11)
Income^a,c^				
<100% Federal Poverty Level (FPL)	26.88	(26.10, 27.66)	50.67	(47.33, 54.02)
101–200% FPL	22.03	(21.29, 22.76)	24.15	(21.30, 27.00)
>200% FPL	51.09	(50.24, 51.95)	25.18	(22.36, 27.99)
Relationship status^a^				
Married	63.72	(62.88, 64.56)	42.73	(39.40, 46.05)
Other	36.28	(35.43, 37.12)	57.27	(53.95, 60.60)
Insurance type at delivery^a^				
Medicaid	38.11	(37.27, 38.96)	61.33	(58.06, 64.60)
Private	55.15	(54.30, 56.01)	32.47	(29.33, 35.62)
Other	6.74	(6.26, 7.21)	6.20	(4.56, 7.84)
Depressive symptoms 3months before pregnancy^a^				
No	85.63	85.02 86.24	56.68	53.34 60.02
Yes	14.37	13.76 14.98	43.32	39.98 46.66

a Rao–Scott *p*-value < .001.

b Rao–Scott *p*-value < .05.

c FPL depends on income and household size. Income was reported in ranges. Respondents’ %FPL was calculated using the midpoint of their reported income range, and their reported number of dependents.

**TABLE 2:** Prevalence of depressive symptoms

	No disability reported		At least one disability	
	Column percent	95% confidence interval	Row percent	95% confidence interval
Antenatal depressive symptoms*				
No	86.52 85.93 87.11	56.88	53.56	60.21
Yes	13.48 12.89 14.07	43.13	39.80	46.45
Postpartum depressive symptoms*				
No	87.92 87.36 88.48	66.44	63.24	69.64
Yes	12.08 11.52 12.64	33.56	30.35	36.76

* Rao–Scott chi-square *p*-value < 0.0001.

**TABLE 3:** Multivariable logistic regression model for depressive symptoms during pregnancy (*N* = 37,715)

Characteristic	Odds ratio	95% CI		*p*-value
Disability status (ref = no)				
**At least one disability**	**2.43**	**1.97**	**2.99**	**<.0001**
Maternal age (ref = 25–34)				
<20	1.17	0.82	1.65	.39
20–24	1.12	0.94	1.32	.20
35+	0.92	0.78	1.07	.26
Maternal education (ref = bachelors+)				
<High school	1.06	0.81	1.39	.68
High School	1.12	0.91	1.38	.28
Some college	1.14	0.95	1.36	.15
Race (ref = white)				
Asian	0.74	0.51	1.07	.11
Black	1.15	0.97	1.38	.11
**Hispanic**	**0.72**	**0.59**	**0.88**	**.001**
Other/Mixed	0.80	0.62	1.03	.084
Income (ref = >200% FPL)				
**<100% FPL**	**1.59**	**1.29**	**1.96**	**<.0001**
**101%−200%**	**1.41**	**1.16**	**1.71**	**.0005**
Marital status (ref = married)				
**Other**	**1.34**	**1.14**	**1.58**	**.0004**
Insurance type (ref = Private)				
Medicaid	1.00	0.83	1.20	.99
Other	1.18	0.89	1.55	.25
Depressive symptoms 3 m before pregnancy (ref = no)				
**Symptoms present**	**33.57**	**29.63**	**38.03**	**<.0001**

Bold values are statistically significant.

**TABLE 4:** Multivariable logistic regression model for depressive symptoms in the postpartum period (*n* = 37,762)

Characteristic	Odds ratio	95% CI		*p*-value
Disability status (ref = no)				
**At least one disability**	**2.14**	**1.80**	**2.54**	**<.0001**
Maternal age (ref = 25–34)				
**<20**	**1.33**	**1.03**	**1.73**	**.03**
**20–24**	**1.17**	**1.02**	**1.35**	**.03**
**35+**	**0.83**	**0.72**	**0.97**	**.02**
Maternal Education (ref = Bachelors+)				
**<High school**	**1.42**	**1.14**	**1.78**	**.002**
**High school**	**1.22**	**1.02**	**1.46**	**.03**
**Some college**	**1.19**	**1.01**	**1.39**	**.03**
Race (ref = white)				
**Asian**	**2.56**	**2.04**	**3.21**	**<.0001**
**Black**	**1.44**	**1.25**	**1.66**	**<.0001**
Hispanic	1.06	0.89	1.25	.53
**Other/Mixed**	**1.36**	**1.06**	**1.74**	**.01**
Income (ref =>200% FPL)				
**<100% FPL**	**1.25**	**1.06**	**1.48**	**.001**
101–200%	1.14	0.98	1.34	.09
Marital status (ref = married)				
Other	0.99	0.86	1.13	.88
Insurance type (ref = Private)				
**Medicaid**	**1.21**	**1.05**	**1.41**	**.01**
Other	0.87	0.68	1.10	.24
History of depression (ref = no)				
**Depression before and/or during pregnancy**	**3.91**	**3.50**	**4.38**	**<.0001**

Bold values are statistically significant.

## Data Availability

The data that support the findings of this study are openly available via a data application to PRAMS at https://www.cdc.gov/prams/prams-data/researchers.htm.
